# Temporal Control of the WNT Signaling Pathway During Cardiac Differentiation Impacts Upon the Maturation State of Human Pluripotent Stem Cell Derived Cardiomyocytes

**DOI:** 10.3389/fmolb.2022.714008

**Published:** 2022-03-24

**Authors:** Chantelle Tsoi, Ruixia Deng, Maxwell Kwok, Bin Yan, Carrie Lee, Hung Sing Li, Chloe Ho Yi Ma, Ruibang Luo, Kam Tong Leung, Godfrey Chi-Fung Chan, Larry Ming-cheung Chow, Ellen N. Poon

**Affiliations:** ^1^ Centre for Cardiovascular Genomics and Medicine, Lui Che Woo Institute of Innovative Medicine, The Chinese University of Hong Kong (CUHK), Shatin, Hong Kong SAR, China; ^2^ Hong Kong Hub of Paediatric Excellence (HK HOPE), CUHK, Shatin, Hong Kong SAR, China; ^3^ Department of Applied Biology and Chemical Technology, The Hong Kong Polytechnic University, Hung Hom, Hong Kong SAR, China; ^4^ Department of Medicine and Therapeutics, CUHK, Shatin, Hong Kong SAR, China; ^5^ Department of Computer Science, Faculty of Engineering, The University of Hong Kong, Pokfulam, Hong Kong SAR, China; ^6^ Department of Paediatrics, CUHK, Shatin, Hong Kong SAR, China; ^7^ The School of Biomedical Sciences, CUHK, Shatin, Hong Kong SAR, China; ^8^ Department of Pediatrics and Adolescent Medicine, The University of Hong Kong, Pokfulam, Hong Kong SAR, China

**Keywords:** hPSC-CMs, cardiac maturation, cardiac differentiation, mitochondria, human pluripotent stem cell derived cardiomyocytes, wnt signalling pathway

## Abstract

Inefficient differentiation and insufficient maturation are barriers to the application of human pluripotent stem cell (hPSC)-derived cardiomyocytes (CMs) for research and therapy. Great strides have been made to the former, and multiple groups have reported cardiac differentiation protocol that can generate hPSC-CMs at high efficiency. Although many such protocols are based on the modulation of the WNT signaling pathway, they differ in their timing and in the WNT inhibitors used. Little is currently known about whether and how conditions of differentiation affect cardiac maturation. Here we adapted multiple cardiac differentiation protocols to improve cost-effectiveness and consistency, and compared the properties of the hPSC-CMs generated. Our results showed that the schedule of differentiation, but not the choice of WNT inhibitors, was a critical determinant not only of differentiation efficiency, which was expected, but also CM maturation. Among cultures with comparable purity, hPSC-CMs generated with different differentiation schedules vary in the expression of genes which are important for developmental maturation, and in their structural, metabolic, calcium transient and proliferative properties. In summary, we demonstrated that simple changes in the schedule of cardiac differentiation could promote maturation. To this end, we have optimized a cardiac differentiation protocol that can simultaneously achieve high differentiation efficiency and enhanced developmental maturation. Our findings would advance the production of hPSC-CMs for research and therapy.

## Introduction

Human pluripotent stem cells (hPSCs) including both embryonic stem cells (ESCs) and induced pluripotent stem cells (iPSCs) have unlimited self-renewal capacity, and their differentiation capability has tremendous potential to advance human cardiac research and therapy by providing an unlimited source of cardiomyocytes (CMs) for transplantation, and as models to investigate heart development and disease (Lu et al., 2017; Musunuru et al., 2018; Paik et al., 2020). However, one of the obstacles to the application of hPSC cardiac cultures is the concurrent differentiation into non-CMs lineages (Ban et al., 2017). The presence of poorly defined non-CMs compromises the safety of hPSC-CMs in cell therapy and leads to inconsistency in disease modeling and drug screening (Biendarra-Tiegs et al., 2020). Another longstanding issue that has greatly limited the use of hPSC-CMs is their poor recapitulation of the adult cardiac phenotype (Robertson et al., 2013; Guo and Pu, 2020). Human PSC-CMs are commonly considered to be developmentally immature: they have disorganized sarcomeres, sparse and poorly-developed mitochondria, preferentially use glycolysis rather than fatty acid oxidation as fuel, inefficient Ca^2+^ transient and electrophysiological properties, along with a transcriptomic and proteomic profile similar to embryonic/fetal, but not adult heart tissue (Poon et al., 2013; Robertson et al., 2013; Bedada et al., 2014; Poon et al., 2015; Chen et al., 2016; Paik et al., 2020). The developmental immaturity of hPSC-CMs limits their ability to mimic the (patho)physiological functions of adult CMs (Biendarra-Tiegs et al., 2020; Poon et al., 2020).

Directed cardiac differentiation of hPSCs has been extensively investigated over the past decade, and considerable improvement has been made in increasing the yield and efficiency of cardiac differentiation protocols. Many protocols nowadays utilize small molecules to drive cardiac differentiation by manipulating the WNT signaling pathway (Lian et al., 2012; Kempf and Zweigerdt, 2018). These protocols usually start with the inhibition of glycogen synthase kinase 3 using CHIR-99021 to induce mesoderm differentiation. This will be followed, in some protocols, by a brief period of culture in media alone, after which the WNT signaling is repressed to drive cardiac specification. Using this strategy, Burridge *et al.* developed a cost-effective differentiation strategy employing a chemically defined platform to produce cardiac cultures containing up to 95% CMs in 11 hiPSC lines (Burridge et al., 2014). Promising results have also been achieved with protocols developed by Hamad *et al.* and Bhattacharya *et al.* with up to 98% efficiency (Bhattacharya et al., 2014; Hamad et al., 2019). These differentiation protocols differ in the temporal control of the WNT signaling pathway and the choice/combinations of WNT inhibitors used. While multiple protocols appear to be successful in generating highly pure hPSC-CM cultures, little is known about whether and how these cultures differ. Here we adapted and optimized protocols previously shown to generate highly pure hPSC-CM cultures and compared the developmental state of the cells generated. We found that schedule of differentiation, but not the choice/combinations of WNT inhibitors, significantly affected the differentiation and maturation of hPSC-CMs. Two of the schedules tested generated CMs with comparable purity of >80%, but the cells differed in the expression of genes important for cardiac maturation, and in their structural, metabolic and proliferative properties. This demonstrate that the temporal control of the WNT signaling pathway during differentiation can regulate the maturation stage of derived hPSC-CMs.

## Materials and Methods

### Human PSC Culture

Undifferentiated hESC (H7: WiCell) and hiPSCs [MD1-C16 (Poon et al., 2020) and SCVI 79: SCVI biobank, abbreviated as 79C1] were cultured on Matrigel-coated (hESC-Qualified, Corning) 6-well plates and maintained in 37°C incubators with 5% CO_2_. Cells were cultured in E8 medium (2ml/well) with supplements (Thermo Fisher Scientific) and medium was refreshed every day. hPSCs were passaged at ∼70% confluency, usually around 3-4 days after seeding. On the day of passage, cells were first rinsed with DMEM/F12 (Thermo Fisher Scientific) and dissociated by incubating in accutase (1ml/well) (Thermo Fisher Scientific) at 37°C for 5 mins. Doubling time was calculated and used to determine initial seeding density for each cell line. Cells were seeded on Matrigel-coated 12-well plates in E8 medium supplemented with Y27632 inhibitor (10 µM; Selleckchem) to ensure cell survival. E8 medium was refreshed without Y27632 inhibitor within 24 h.

### Cardiac Differentiation

We initiated cardiac differentiation when the confluency reached 70–90% 3-4 days after seeding. Cardiac differentiation was performed using the chemically defined medium (CDM3) as reported previously (Burridge et al., 2014).

**FIGURE 1:**
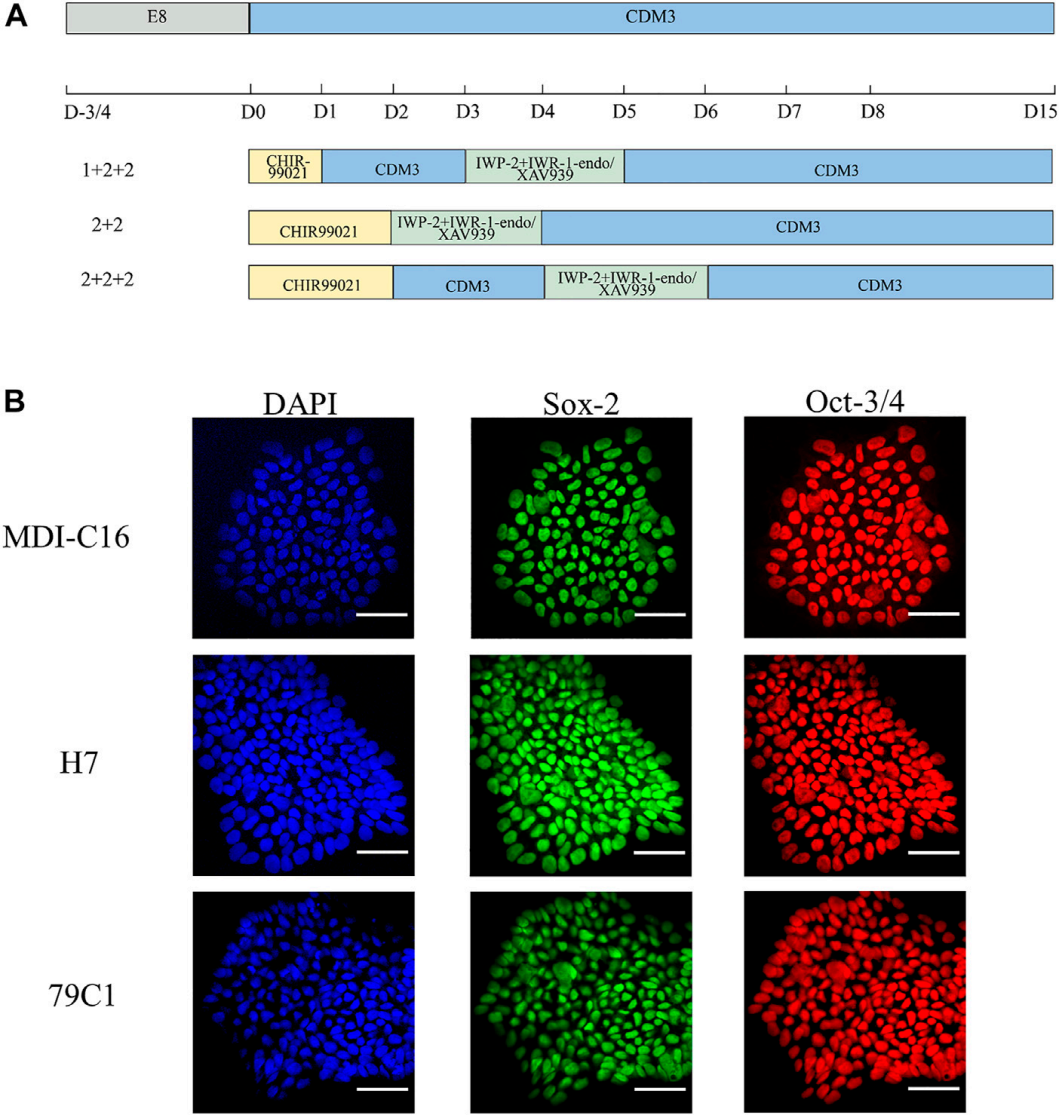
Schematic of cardiac differentiation and characterization of hPSCs. **(A)** Schematic of hPSC cardiac differentiation in chemically defined medium (CDM3) from Day 0–15 following different schedules (1 + 2 + 2, 2 + 2, 2 + 2 + 2). **(B)** Confocal images of pluripotency markers in undifferentiated hPSCs. DAPI for nuclei (blue), Sox-2 (green), Oct-3/4 (red). Scale bar, 50 µm.

In order to understand the effect of timing and WNT inhibitors on cardiac differentiation efficiency, three induction schedules and two combinations of WNT inhibitors were investigated in this study (as shown in Figure 1A).

We first used CHIR-99021 (6 µM; Cayman Chemical) to initiate differentiation on day (D)0. In the “1 + 2 + 2” differentiation protocol, CDM3 medium was refreshed the following day from D1 to D3, followed by the combination of IWP-2 (5 µM; APExBIO) with either IWR-1-endo (5 µM; Cayman Chemical) or XAV939 (5 µM; Cayman Chemical) applied from D3 to D5. For the “2 + 2” differentiation protocol, a 2-day incubation in CHIR-99021 was followed by another 2-day incubation with either IWP-2 + IWP-1-endo or IWP-2 + XAV939. The “2 + 2 + 2” protocol started with CHIR-99021 treatment for 2 days, but with CDM3 medium refreshed from D2 to D4 before addition of either IWP-2 + IWP-1-endo or IWP-2 + XAV939 from D4 to D6. CDM3 medium were changed every 2 days until D15 to D18, when cells were dissociated for use of analysis by flow cytometry or cell passaging.

### Analysis of CMs by Flow Cytometry

On the day of analysis, cells were washed with Dulbecco’s phosphate buffered saline no calcium, no magnesium (DPBS-/-) (Thermo Fisher Scientific) and dissociated with Trypsin-EDTA (0.05%) (Thermo Fisher Scientific) at 37°C for 5–15 min (1ml/well). Cells were then neutralized with an equal volume of RPMI medium (Thermo Fisher Scientific) supplemented with 10% fetal bovine serum (FBS) (HyClone) and centrifuged at 300 × g for 4 min at 4°C. Samples were prepared as previously described (Burridge et al., 2014). An antibody against cardiac troponin T (TNNT2) (Abcam) (Waas et al., 2019) or mouse IgG1 isotype control (BioLegend) were applied for 1 h at 4°C. Cells were washed once with wash buffer (0.5% BSA and 0.1% Triton X-100 in DPBS-/-). Goat anti-Mouse IgG1 secondary antibody (Thermo Fisher Scientific) was then applied, then washed once with wash buffer. Cells were analyzed using the CytoFLEX Flow Cytometer (Beckman Coulter) or Attune NxT Flow Cytometer (Thermo Fisher Scientific). The results were analyzed with the software FlowJo 10.6.2. Populations were gated against <2% of the isotype control.

### Immunofluorescence Staining

Glass cover slips were coated with Matrigel at least 1 h prior to plating. Cells were dissociated and plated onto the Matrigel-coated cover slips and cultured until the required time point. Cells were washed with phosphate-buffered saline (PBS), fixed in 4% PFA (prepared with PBS) for 15 min at room temperature and permeabilized with 0.1% Triton X-100 in PBS for 10 min at room temperature. Following blocking with 10% FBS in PBS for 30 min at room temperature, cells were incubated in primary antibodies prepared in 10% FBS in PBS at 4°C overnight. After washing with PBS, cells were incubated in secondary antibodies at 1:1,000 dilution for 1 h at room temperature, and washed in PBS for 3 times. The cover slips were then mounted on glass slides with ProLong Diamond Antifade Mountant with DAPI (Thermo Fisher Scientific). Cells were imaged using the Leica DM6B Motorized Upright Microscope or Nikon A1 Confocal Microscope.

### Quantification of Cardiac Myosin Subtype Expression

Cells were stained with antibodies against MLC2A (Synaptic Systems) and MLC2V (ProteinTech) as described above. 40X images were taken using the Leica DM6B Motorized Upright Microscope with 5-6 fields from each sample, >300 cells per sample. The proportion of MLC2A and MLC2V positive cells were counted using the ImageJ software.

### Quantitative Real-Time PCR

TRIzol Reagent (Thermo Fisher Scientific) was used to extract total RNA according to the manufacturer’s protocol. cDNA was synthesized with PrimeScript RT Master Mix kit (TaKaRa). Quantitative SYBR Green RT-PCR was then performed. Each sample was run using three technical replicates. RNA levels were normalized to that of B2M. (See Supplementary Table S1 for primer sequences).

### Determination of Mitochondria Abundance and Fatty Acid Uptake by Flow Cytometry

Cells were first seeded onto 12-wells at a density of around 200k/well and medium was changed every 2-3 days. On day 30 ± 3, CMs were stained with the MitoTracker deep red (MTDR) dye (200 nM; Thermo Fisher Scientific) and a fluorescently labelled palmitate analogue BODIPY FL C16 (10 µM; Thermo Fisher Scientific) for 25 min. The cells were dissociated, washed and resuspended in Hank’s buffered saline solution (HBSS) containing 10 µM Y27632 inhibitor, 25 mM HEPES (Thermo Fisher Scientific), and 5% FBS. DAPI staining (2.5 µM) was also included to help gate out dead cells during flow cytometry. Cells were then loaded onto the Attune NxT Flow Cytometer (Thermo Fisher Scientific) and analyzed. BODIPY Fl C16 and MTDR staining were calculated as the median FITC-A and APC-A, respectively.

### Seahorse Metabolic Flux Assay

Oxidative respiration was assessed with the mito-stress assay, which measures the oxygen consumption rate (OCR), using a Seahorse extracellular flux analyzer (XFe-96) (Agilent Technologies, CA, United States) as per manufacturer’s protocols. CM cultures were seeded at approximately 1 × 104 cells/well on a Seahorse 96-well XF Cell Culture Microplate (Agilent). The mito-stress assay involved the sequential application of oligomycin (1 μM), FCCP (1 μM) and antimycin A/rotenone (0.5 μM) during which the OCR was measured. Basal and maximal respiration and spare respiratory capacity were calculated using the Wave software (Agilent).

### Proliferation Assay

Human PSC-CMs were stained with anti-Ki67 antibody (Abcam) as described above. EdU incorporation assay was performed using the Click-iT™ Plus EdU Cell Proliferation Kit (Thermo Fisher Scientific) as per manufacturer’s instructions. The number of positive nuclei was quantified using the ImageJ software. >300 cells were counted per batch of cells.

### Measurement of Ca^2+^ Transient Properties

Cells were stained with calcium indicator Calbryte 590-AM (5 μM; AAT Bioquest) and 0.02% Pluronic F-127 (Biotium) in CDM3 at 37°C for 30 mins, and washed in warm CDM3 media twice. Fluorescence videos of calcium transients were taken at 100 frames per second using Leica THUNDER imaging system with 37°C and 5% CO_2_ incubation. Individual cells from the videos were manually segmented and calcium transient traces were extracted with Leica LAS X software. The traces were analyzed with the software MATLAB R2019b using CalTrack code (Psaras et al., 2021).

### miRNA-Target Analysis

Two resources were used for miRNA-target prediction. mirTarBase is a database of miRNA target genes that is supported by experimental validation (Huang et al., 2020). Second, miRecords, a web-based platform, contains the predicted binding target genes of miRNAs based on 11 computational methods (Xiao et al., 2009).

### Transcription Factor Binding Target Gene Analysis

To identify target genes of cardiac TFs (GATA4,6, HAND1,2, NKX2-5, TBX5, and HEY2), we searched for binding sites conserved on human and mouse promoters using PWMSCAN (Wang et al., 2007). This method conducts computational identification of the binding sites by scanning promoter sequences using Position Weight Matrices of TF binding motifs. The putative binding sites were further filtered based on conservation scores between human and mouse genomes. Promoter analyses were limited to locations 2000 bp upstream and 500 bp downstream from the transcriptional start site.

### Statistical Analysis

Statistical analysis was performed using ratio *t*-test for comparisons between two groups. One-way ANOVA and Two-way ANOVA for one-factor and two-factor comparisons were used among three groups respectively. Significant differences were defined as *p < 0.05, **p < 0.01, and ***p < 0.005, respectively, and data are presented as mean ± S.E.M.

## Results

### The Temporal Control of WNT Manipulation Determined Efficiency and Yield of Cardiac Differentiation

To achieve robust and cost-effective cardiac differentiation, we combined critical elements from three protocols previously shown to generate hPSC-CMs at high efficiency (Bhattacharya et al., 2014;Burridge et al., 2014;Hamad et al., 2019). We first adapted the protocols onto the CDM3 media used by Burridge *et al.*, which is chemically-defined and is more cost-effective than the traditional B27 media used by Hamad *et al.* and Bhattacharya *et al.* (Bhattacharya et al., 2014;Burridge et al., 2014;Hamad et al., 2019). Our second modification is the simultaneous application of two WNT inhibitors, previously suggested by Hamad *et al.* to minimize batch-to-batch variations (Hamad et al., 2019). Based on the three original publications, we devised six cardiac differentiation protocols that differed in their schedules and in the WNT inhibitors used (Figure 1A). Three schedules were chosen (“1 + 2 + 2”, “2 + 2”, and “2 + 2 + 2”), corresponding to each of the three original publications and differ in the duration of CHIR-99201 treatment, and the presence of a rest period between the application of CHIR-99201 and WNT inhibitors. For each schedule, we used two combinations of WNT signaling inhibitors: IWP-2 + IWR-1-endo and IWP-2 + XAV939. To make sure that our results can be generalized, three hPSC lines were tested including one “normal” hESC (H7), one normal hiPSC (MDI-C16) and one “disease” hiPSC cell line, in this case 79C1 derived from a patient with doxorubicin-induced cardiomyopathy (Burridge et al., 2016).

We first confirmed the pluripotency of the three cell lines by showing that they were positive for well-established markers Sox-2 and Oct-3/4 (Figure 1B). Next, we optimized the cell density at the time of induction. Cardiac differentiation at 50% density using the six protocols resulted in cultures that showed no beating, while differentiation at 70 and 90% density produced cultures with similar beating behavior in all three cell lines (data not shown). As a result, all inductions onwards were carried out using cell density at a range of 70–90% at the start of differentiation.

**FIGURE 2:**
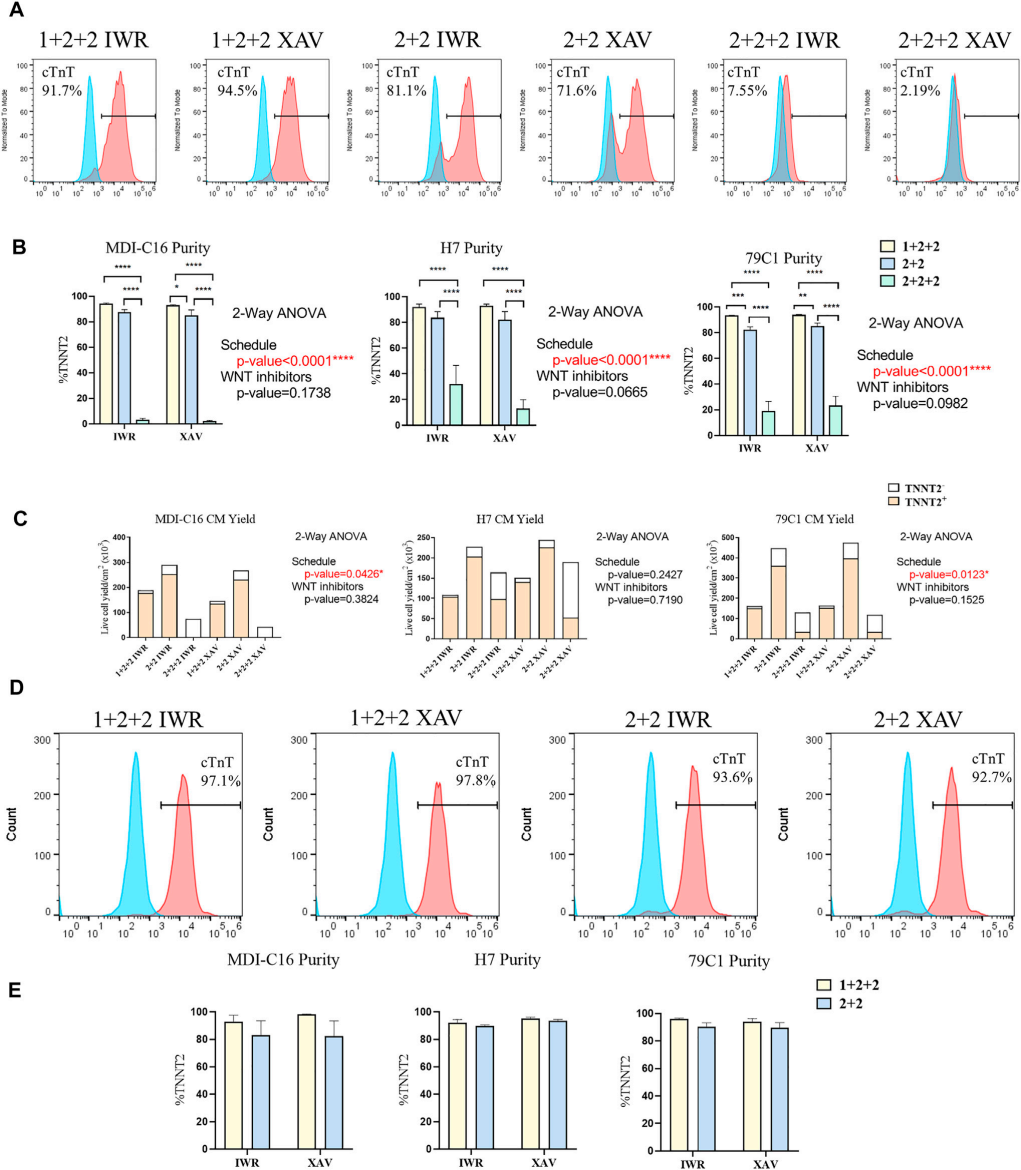
Temporal control of the WNT signaling pathway determined the efficiency and yield of cardiac differentiation. Human PSCs were differentiated using different differentiation protocols. **(A)** Representative flow cytometric analysis of MD1-C16 derived CMs and **(B)** graphs showed the proportion of TNNT2^+^ cells on day 15 of differentiation. Blue and red histograms represent isotype and TNNT2^+^ staining respectively (*n* = 5). **(C)** Live cell yield of hPSC-CM cultures presented as the average of independent experiments (*n* = 3–4). **(D)** Representative flow cytometric analysis of MD1-C16 derived CMs and **(E)** graphs showed the proportion of TNNT2^+^ cells on day 30 of differentiation. (*n* = 3).

Human PSC-CM cultures generated using both the “1 + 2 + 2” and “2 + 2” protocols started spontaneous contraction around day 8-9. Both protocols generated CMs with similar morphology and formed a “net-like” monolayer when beating is observed (Supplementary Figure S1, Supplementary Videos S1, S2). Conversely, few beating clusters were observed in “2 + 2 + 2” cultures. To quantify the cardiac differentiation efficiency of the six protocols, we performed flow cytometric analysis of cardiac troponin T (TNNT2), an established marker of CMs on D15 of differentiation (Figure 2). The schedule of differentiation, but not the choice of WNT inhibitors significantly impact upon differentiation efficiency across all cell lines. In the MDI-C16 hiPSC line, the “1 + 2 + 2” protocol yielded the highest percentage of TNNT2 positive cells (93.7 ± 1.1% and 92.6 ± 0.9% for IWP-2 + IWR-1-endo and IWP-2 + XAV939, respectively), followed by the “2 + 2” protocol (87.1 ± 2.5% and 84.6 ± 4.7% for IWP-2 + IWR-1-endo and IWP-2 + XAV939, respectively) (Figures 2A,B). However, the “2 + 2 + 2” protocol generated few CMs (2.8 ± 1.6% and 1.9 ± 0.9% for IWP-2 + IWR-1-endo and IWP-2 + XAV939, respectively).

We also evaluated the CM yield of the six protocols and found that “1 + 2 + 2” cultures yielded a lower number of CMs (178.2 ± 34.6 × 10^3^ cells/cm^2^ and 135.8 ± 8.0 × 10^3^ cells/cm^2^ for IWP-2 + IWR-1-endo and IWP-2 + XAV939 respectively) than “2 + 2” cultures (253.0 ± 58.8 × 10^3^ cells/cm^2^ and 231.6.6 ± 49.9 × 10^3^ cells/cm^2^ for IWP-2 + IWR-1-endo and IWP-2 + XAV939, respectively), with the lowest CM yield in the “2 + 2 + 2” protocol (3.1 ± 2.5 × 10^3^ cells/cm^2^ and 0.5 ± 0.2 × 10^3^ cells/cm^2^ for IWP-2 + IWR-1-endo and IWP-2 + XAV939, respectively) (Figure 2C).

Since CMs have relatively low proliferation potential, the presence of non-CMs may overwhelm CMs and decrease purity at later stages of differentiation. The purity of cardiac cultures was measured again by flow cytometry at D30 of differentiation. The “1 + 2 + 2” yielded a slightly higher percentage of TNNT2^+^ cells (92.9 ± 4.7% and 98.3 ± 0.29% for IWP-2 + IWR-1-endo and IWP-2 + XAV939, respectively), than “2 + 2” (82.9 ± 10.7% and 82.4 ± 11.1% for IWP-2 + IWR-1-endo and IWP-2 + XAV939, respectively), but differences were not statistically significant (Figures 2D,E). Our results showed that our optimized protocols could generate CMs at high efficiency, and that purity was maintained from D15 to D30.

Overall, our results showed that two out of the three schedules tested could generate CMs at high efficiency, and that temporal control of WNT signaling exert a strong effect on cardiac differentiation. Similar observations were made on the H7 and 79C1 lines (Figures 2B,C,E and Supplementary Figure S2), showing that the trends observed were independent of cell lines. The choice of WNT inhibitors did not significantly affect differentiation, thus we focused our subsequent analysis on cells differentiated using IWP-2 + XAX939. “2 + 2 + 2” protocol gave significantly lower purity and yield, and was therefore eliminated in the study onwards.

### The Temporal Control of WNT Manipulation During Cardiac Differentiation Affected the Expression of Genes Important for Developmental Maturation

**FIGURE 3:**
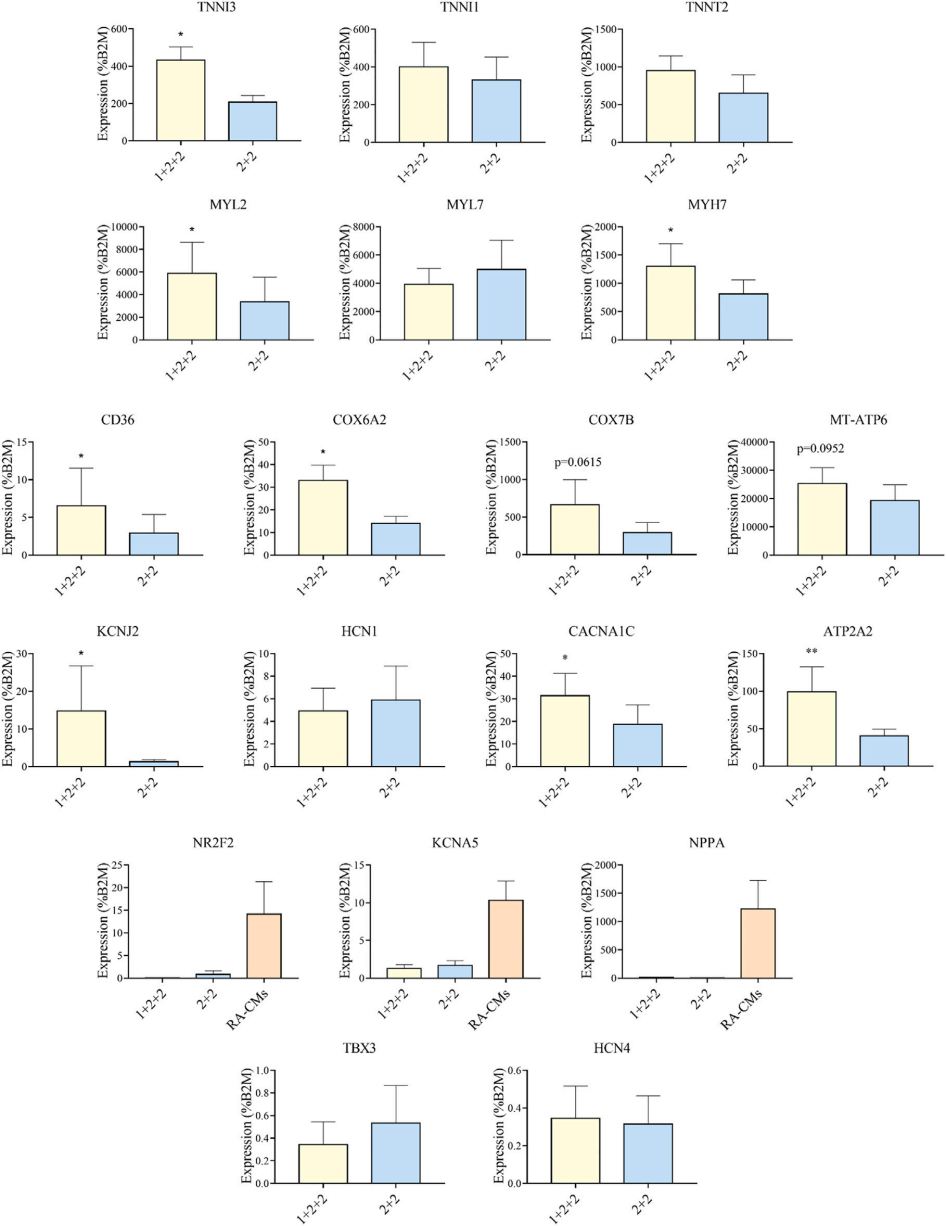
Differentiation schedule affected the expression of genes important for cardiac maturation. RT-qPCR analysis of MDI-C16 hPSC-CMs derived from selected differentiation protocols on day 30 ± 3 of differentiation (*n* = 3–8). CMs differentiated with the addition of retinoic acid (RA-CMs), which has previously been shown to induce atrial specification, were used as positive control for NR2F2, KCNA5 and NPPA.

Given that the “1 + 2 + 2” and “2 + 2” protocols both generated cultures with high efficiency, we asked if resultant hPSC-CMs were different. We performed RT-qPCR to compare the mRNA expression of genes important for structural, metabolic, electrophysiological and Ca^2+^ transient properties in MDI-C16 hPSC-CMs differentiated using the two protocols (Figure 3). Structural genes such as troponin and myosin isoforms are developmentally regulated and their expression are often used to indicate structural maturity (Bedada et al., 2014). Slow skeletal troponin I (TNNI1) and myosin light chain 2A/MLC2A (MYL7) are commonly detected in early stage hPSC-CMs while cardiac troponin I (TNNI3), myosin light chain 2V/MLC2V (MYL2) and *ß* myosin heavy chain (MYH7) expression become predominant as hPSC-CMs mature (Guo and Pu, 2020). We found that “1 + 2 + 2” cultures expressed significantly higher levels of TNNI3 (2.26 ± 0.43 fold), MYL2 (2.79 ± 0.91 fold) and MYH7 (1.63 ± 0.27 fold) compared to “2 + 2” cultures, while TNNI1 and MYL7 levels were not significantly different. TNNT2, a pan cardiac marker, was also found at similar levels, consistent with similar CM purity of both cultures (Figure 2). Next, we evaluated the expression of genes critical for cardiac metabolism. CD36, a fatty acid transporter which is also a marker of hPSC metabolic maturation (Poon et al., 2020), was found to be upregulated (3.85 ± 1.23 fold) in “1 + 2 + 2” cultures. COX6A2, COX7B, and MT-ATP6, which encode subunits of mitochondrial complex IV and ATPase of the electron transport chain, were also significantly increased by 2.38 ± 0.21, or tended to increase by 3.19 ± 0.91 and 1.89 ± 0.55 fold in “1 + 2 + 2” compared to “2 + 2” cultures respectively. As for electrophysiology, we assessed the expression of genes encoding the subunits of IK_1_ (KCNJ2) and I_f_ (HCN1), which are responsible for determining the resting membrane potential in mature CMs, and in automaticity in immature cells, respectively. KCNJ2 (9.74 ± 4.13 fold) was significantly upregulated, while HCN1 level was similar in “1 + 2 + 2” compared to “2 + 2” cultures. The levels of CACNA1C and ATP2A2, which encodes the L-type Calcium channel and SERCA (sarco/endoplasmic reticulum Ca^2+^-ATPase), were also significantly higher by 2.15 ± 0.40 and 2.18 ± 0.37 fold in “1 + 2 + 2” compared to “2 + 2” cultures (Figure 3). Lastly, we assessed the expression of genes commonly found in atrial and pacemaker CMs. The levels of atrial-prevalent genes such as NR2F2 (COUP-TFII), KCNA5 and NPPA, and pacemaker-prevalent genes including TBX3, HCN4 and SHOX2 were either very low (NR2F2, KCNA5, NPPA, TBX3, and HCN4) or undetectable (SHOX2). Expression was not significantly different between the two cultures, suggesting similar sub-type specifications. Similar trends were observed in 79C1 cultures (Supplementary Figure S3).

Our results showed that the temporal control of WNT manipulation during cardiac differentiation greatly affected the expression of genes crucial for cardiac maturation. Specifically, hPSC-CMs differentiated using the “1 + 2 + 2” protocol had an expression pattern suggestive of more advanced structural, metabolic, electrophysiological and Ca^2+^ handling properties.

### The Temporal Control of WNT Manipulation During Cardiac Differentiation Influenced the Structure of hPSC-CMs

**FIGURE 4:**
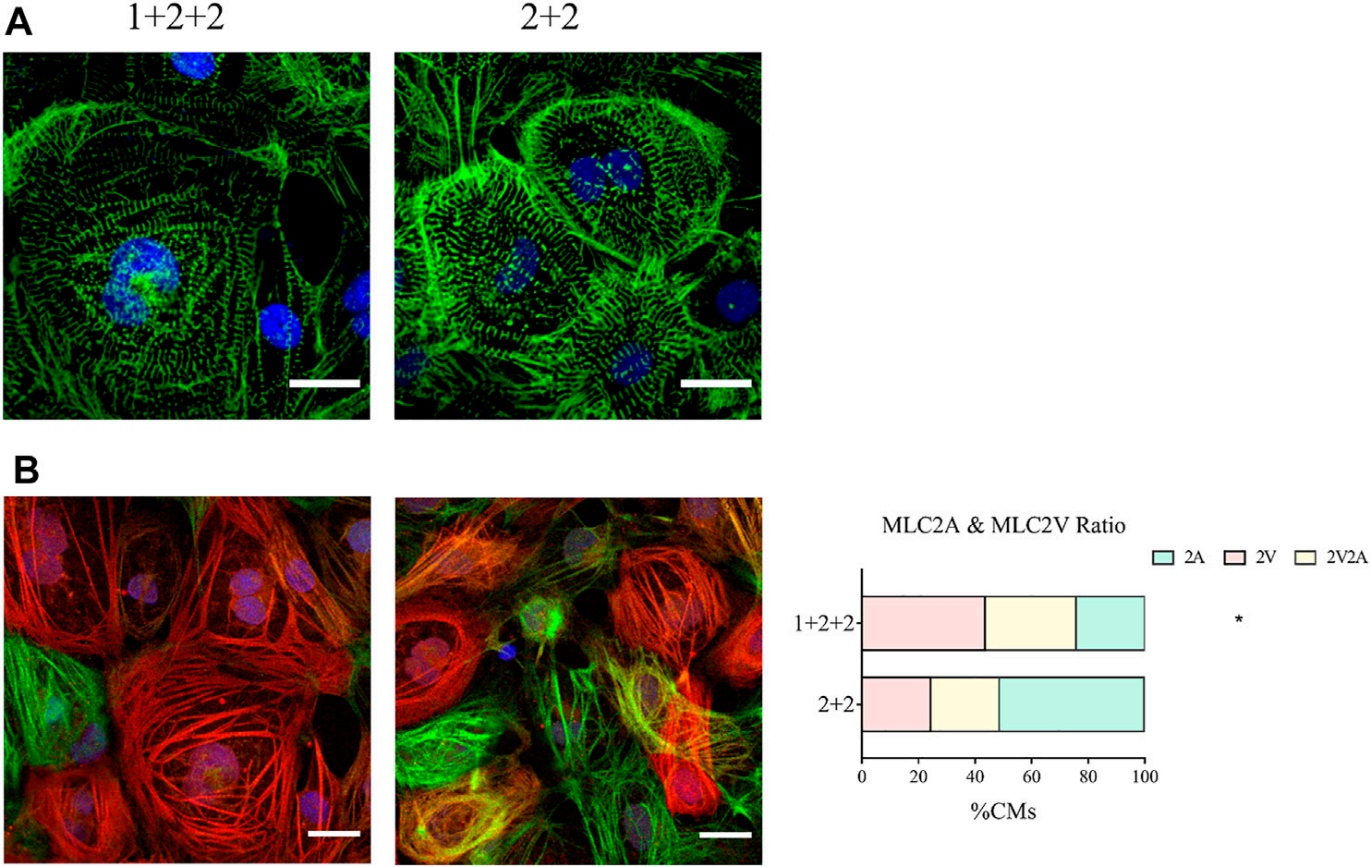
Differentiation schedule influenced the structural properties of hPSC-CMs. Confocal images of **(A)** α-actinin (green) and **(B)** MLC2A (green) and MLC2V (red) in MDI-C16 hPSC-CMs derived from selected differentiation protocols on day 30 of differentiation (*n* = 4 independent experiments). Scale bar, 20 µm. The proportion of MLC2A^+^, MLC2A^+^ MLC2V^+^, and MLC2V^+^ cells in MDI-C16 hPSC-CMs was quantified (*n* = 4 independent experiments, >50 cells per batch).

We next compared the structural properties of “1 + 2 + 2” and “2 + 2” cultures at D30 of differentiation. Immunofluorescence staining of α-actinin revealed a mixture of CMs with disorganized, as well as organized striated sarcomeric structures. “1 + 2 +2” and “2 + 2” cultures were not notably different (Figure 4A).

Our qPCR analysis revealed differential abundance of MLC2V and MLC2A, we thus seek to validate this on the protein level by immunofluorescence staining. Early stage hPSC-CMs are thought to express mostly MLC2A, then they co-express MLC2A and 2V as they gain intermediate maturity, while later stage hPSC-CMs are thought to express primarily MLC2V. Immunofluorescence staining showed that most CMs generated with the “1 + 2 + 2” protocol expressed MLC2V, while MLC2A could be detected in many CMs differentiated with the “2 + 2” protocol (Figure 4B). Quantification of cells expressing MLC2V alone, both MLCV and MLC2A, and MLC2A alone revealed that the “1 + 2 + 2” protocol yielded a significantly higher proportion of cells which expressed MLC2V alone (Figure 4B). These results were generally consistent in all three cell lines (Supplementary Figure S4) and indicated greater structural maturation in “1 + 2 + 2” cultures.

### The Temporal Control of WNT Manipulation During Cardiac Differentiation Impacted Upon the Mitochondrial and Metabolic Maturation of hPSC-CMs

**FIGURE 5:**
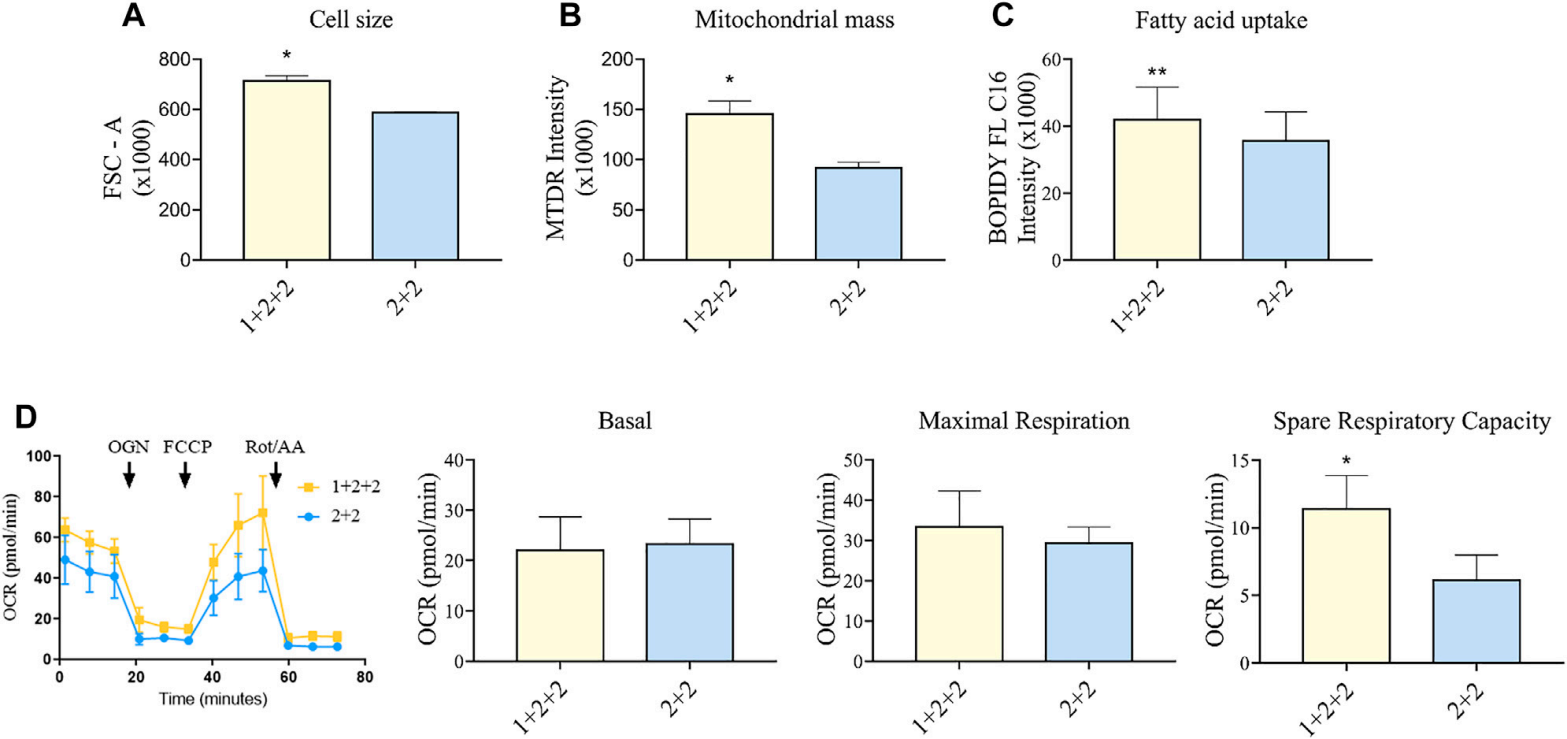
Differentiation schedule impacted upon the mitochondrial and metabolic maturation of hPSC-CMs on day 30 ± 3 of differentiation. **(A)** Forward-scatter (FSC) was measured as a surrogate of cell size in MDI-C16 hPSC-CMs derived from selected differentiation protocols (*n* = 3). **(B)** Mitochondrial abundance was measured using MitoTracker Deep Red staining (*n* = 4). **(C)** Fatty acid uptake was monitored using BODIPY FL C16 staining (*n* = 5). **(D)** Seahorse metabolic flux analysis comparing key metabolic parameters. FCCP, carbonyl cyanide p-trifluoro-methoxyphenyl hydrazone; OCR, oxygen consumption rate; OGN, oligomycin; Rot/AA, rotenone + actimycin (*n* = 5).

While early hPSC-CMs remain small and resemble fetal cardiomyocytes, they become larger when they mature. We evaluated this by quantifying forward-scatter (FSC) which is a surrogate measure of cell size, and showed that “1 + 2 + 2” cultures displayed a significantly greater FSC, and hence are larger, than the “2 + 2” cultures (Figure 5A).

We also sought to determine whether there was a difference in the mitochondrial functionality and metabolism in hPSC-CMs generated using different schedules. As adult CMs primarily rely on mitochondrial oxidative phosphorylation as a means to generate energy (Guo and Pu, 2020), a higher mitochondrial mass is a sign of maturity. Flow cytometric analysis using mitochondrial dye showed that “1 + 2 + 2” cultures had a significantly greater mitochondrial mass (1.60 ± 0.19 fold) compared to “2 + 2” cultures (Figure 5B). This is consistent with higher levels of mitochondrial genes detected by qPCR (Figure 3). A switch to fatty acid oxidation as an energy fuel also signifies metabolic maturation in cells. We assessed this by quantifying the uptake of a fluorescently labeled analogue of palmitic acid. Human PSC-CMs generated using “1 + 2 + 2” protocol showed greater accumulation of this dye (1.18 ± 0.03 fold) compared to those from “2 + 2” (Figure 5C). This is consistent with increased mRNA expression of CD36, a major transporter of long chain fatty acids, in these cultures (Figure 3). Similar results were observed across cell lines (Supplementary Figure S5). Lastly, we performed Seahorse metabolic flux analysis to evaluate the oxidative respiration capacity of our hPSC-CMs. While basal and maximal respiration were similar, “1 + 2 + 2” cultures had a significantly higher spare respiratory capacity compared to “2 + 2” cultures (2.37 ± 0.45 fold), showing that the temporal control of WNT signaling pathway impacted upon the mitochondrial and metabolic function of the cells generated (Figure 5D).

### The Temporal Control of WNT Manipulation During Cardiac Differentiation Influenced the Proliferation of hPSC-CMs

**FIGURE 6:**
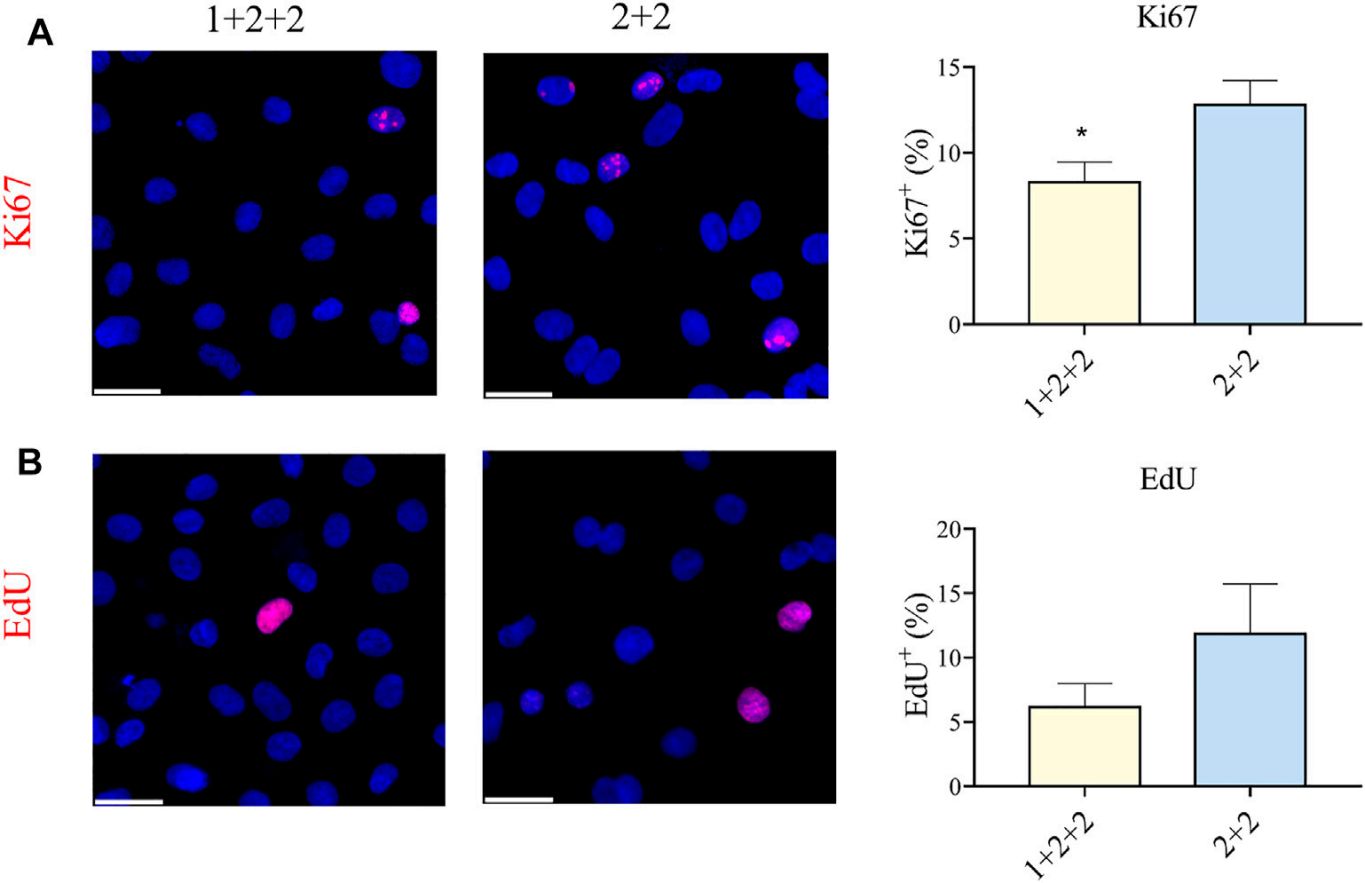
Differentiation schedule influenced the proliferative potential of hPSC-CMs. Human PSC-CMs were assayed for **(A)** Ki67 staining or **(B)** EdU incorporation. The proportion of positive cells were quantified using the ImageJ software (*n* = 8, *n* = 3).

CMs are proliferative during embryonic development, but they exit the cell cycle after birth. Adult CMs are commonly thought to have little proliferative capacity. We measured the proportion of cells positive for Ki67, an established marker of proliferation, and found that “1 + 2 + 2” cultures had a significantly lower proportion of Ki67^+^ CMs compared to “2 + 2” cultures (8.28 ± 1.17 vs. 12.78 ± 1.44%) (Figure 6A). These were corroborated by EdU incorporation, a functional assay for proliferation, which also demonstrated a qualitatively lower level of proliferation in “1 + 2 + 2” cultures (Figure 6B). These results are again consistent with a more mature phenotype in “1 + 2 + 2” cultures.

### The Temporal Control of WNT Manipulation During Cardiac Differentiation Impacted Upon the Ca^2+^ Transient Properties in hPSC-CMs

**FIGURE 7:**
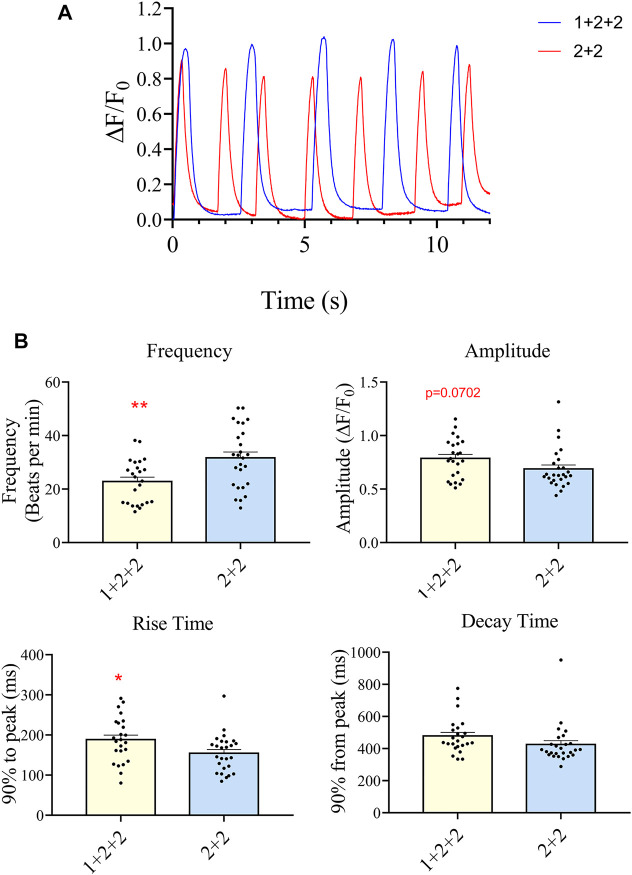
Differentiation schedule impacted upon the Ca^2+^ transient properties of hPSC-CMs. Ca^2+^ transient properties were monitored using the Calbryte dye. **(A)** Representative tracings are shown. **(B)** The average frequency, amplitude, rise (from time = 0, time to 90% peak) and decay time (from peak, time to 10% of peak) were quantified (”1 + 2 + 2” *n* = 24, “2 + 2” *n* = 26).

We next evaluated the Ca^2+^ transient properties of “1 + 2 + 2” and “2 + 2” hPSC-CMs using the Calbryte 590 calcium indicator (Figures 7A, B). H7-CMs generated using the “1 + 2 + 2” protocol had a significantly lower average frequency of cytosolic Ca^2+^ release than those from the “2 + 2” protocol (22.8 ± 1.7 vs. 31.7 ± 2.2 beats/min), consistent with a more mature state. The amplitude of Ca^2+^ transients tended to increase, and this was accompanied by a modest increase in the rise time (188.3 ± 11.5 vs. 154.6 ± 9.2 ms) and similar decay time.

### Potential Regulation of Differential mRNA Expression

**FIGURE 8:**
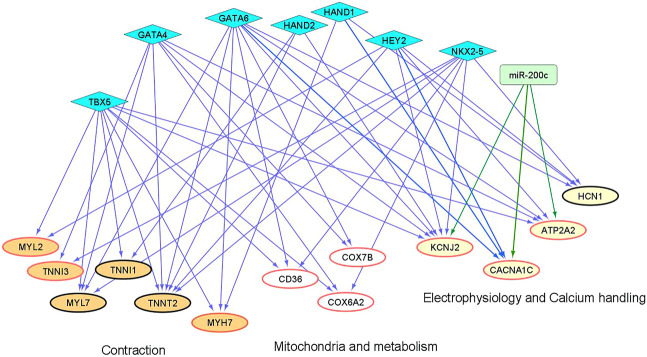
An inferred cardiac gene regulatory network underlying the transcriptomic differences between differentiation protocols. Two sets of regulators are displayed, miRNAs (miR-200c in rectangle) and cardiac TFs (in diamonds). The proposed regulatory relationships between miR-200c or TFs and target genes are indicated by blue and green edges, respectively. Oval nodes correspond to target genes involved in various cardiac biological processes: Orange—contraction; White—mitochondria/metabolism; Yellow—electrophysiology and calcium handling. Up-regulated genes are shown with red borders.

We and others have shown that microRNAs (miRs) and cardiac transcription factors (TFs), such as miR-200c, and a set of GATA4/6, NKX2-5, TBX5, HEY2, and HAND1/2 can cooperatively regulate the expression of genes critical for cardiac development and function (Poon et al., 2011;Poon et al., 2013;Cerutti et al., 2014;Rojas-Munoz et al., 2014;Kathiriya et al., 2015;Poon et al., 2018). To investigate if these regulatory factors could contribute to the differential expression of genes involved in maturation between “1 + 2 + 2” and “2 + 2” cultures (Figure 3), we identified binding sites of the cardiac TFs on the promoters of these ‘maturation genes’. Genes targeted by miR-200c was extracted from mirTarBase and miRecords, two databases containing experimental reports and computational prediction, respectively. As shown in Figure 8, conserved binding sites of the cardiac TFs were identified on promoters of these maturation genes, providing evidence for a transcriptional regulatory role of these cardiac TFs. We also predict that miR-200c could regulate “maturation genes” including ATP2A2, CACNA1C, and KCNJ2 as validated in our previous experiments (Poon et al., 2018). Our analysis revealed potential co-regulation of maturation genes by multiple factors. In particular, KCNJ2 is predicted to be regulated by all six TFs and miR-200c. This finding suggests that crosstalk of cardiac TFs and miR200c may contribute to differential expression of the maturation genes among our “1 + 2 + 2” and “2 + 2” cardiac cultures.

## Discussion

During the last decade, researchers have focused on promoting cardiac differentiation with high efficiency. As a result of such intense efforts, many groups have reported cardiac differentiation protocols that can efficiently generate CMs across many cell lines. Here we adapted and optimized protocols previously shown to generate highly pure hPSC-CM cultures, and compared the properties of the CMs generated. Two of the differentiation schedules tested generated CMs with high efficiency but the differentiated CMs differed in the expression of genes important for cardiac maturation, and in their functional properties. Thus temporal control of the WNT signaling pathway during early differentiation significantly affect not only cardiac differentiation efficiency, but also the maturation of hPSC-CMs.

The manipulation of the WNT signaling pathway has become the basis of directed cardiac differentiation. Highly efficient protocols have been developed by Bhattacharya *et al.*, Hamad *et al.* and Burridge *et al.*, who each reported the generation of hPSC-CMs at >80% purity (Bhattacharya et al., 2014; Burridge et al., 2014; Hamad et al., 2019). These protocols differed in their differentiation schedule, choice of WNT inhibitors and basal media. We optimized these protocols by adapting them to the CDM3 media, which is chemically defined and more cost-effective than the traditional B27 media (Burridge et al., 2014). With the “1 + 2 + 2” and “2 + 2” protocols, we were able to generable CMs in CDM3 media at high yield and consistency in line with the parent protocols. The “2 + 2 + 2” protocol was generally unsuccessful, and this may relate to the use of CDM3, rather than B27 media as was originally proposed (Bhattacharya et al., 2014). Burridge *et al.* has previously suggested that the optimal timing of WNT manipulation is affected by the choice of basal media, and this may explain the discrepancies between our results and those reported by Bhattacharya *et al.* A further modification involves the simultaneous application of two inhibitors targeting different components of the WNT signaling pathway, which was reported to reduce batch-to-batch variations (Hamad et al., 2019). Two combinations of WNT inhibitors were tested in this study. IWP-2 is a porcupine WNT inhibitor that was used in the original report showing that cardiac differentiation could be achieved via the modulation of the WNT signaling pathway (Lian et al., 2012), and was shown to be effective in numerous publications (Burridge et al., 2014; Zhang et al., 2015). Here we used it in combination with either XAV-939 or IWR-1-endo. XAV-939 was used by Hamad *et al.*, while IWR-1-endo was used by Bhattacharya *et al.* (Bhattacharya et al., 2014; Hamad et al., 2019). XAV-939 and IWR-1-endo both inhibit WNT signaling by targeting tankyrase. Therefore, it is not surprising that the IWP-2 + XAV-939 and IWP-2 + IWR-1-endo combinations generated CMs at similar purity and yield.

Many strategies have been devised to promote the maturation of hPSC-CMs (Karbassi et al., 2020), and they include long-term culture (Kamakura et al., 2013), 3-dimentional (D) culture (Ronaldson-Bouchard et al., 2018; Ronaldson-Bouchard and Vunjak-Novakovic, 2018; Gu et al., 2019), electrical stimulation (Ronaldson-Bouchard et al., 2018), the use of specific small molecules and supplementation (Yang et al., 2014; Poon et al., 2015; Yang et al., 2019), culture on or encapsulated in specific substrates (Chen et al., 2017; Wang et al., 2017) and the isolation of mature hPSC-CM subpopulation using cell surface markers (Poon et al., 2020; Boheler and Poon, 2021). The above strategies mostly involve exposing differentiated hPSC-CMs to conditions that promote maturation. How early events during differentiation affect the maturation stage of the resultant cells is not clear. Previous work has compared hPSC-CMs differentiated from parallel 2- and 3D cultures. Zhang *et al.* found that 2D monolayer cultures yielded hPSC-CMs at higher purity, but differentiation progressed at similar kinetics relative to 3D embryoid body cultures (Zhang et al., 2015). The resultant cells also have comparable electrophysiological and Ca^2+^ transient properties. Recent work has shown that hydrogel can support the differentiation and maintenance of hPSC-CMs (Kerscher et al., 2016; Chen et al., 2017). Kerscher *et al.* compared 3D hydrogel structures with 2D cultures and showed similar gene expression and behavior (Kerscher et al., 2016). Conversely, Branco *et al.* suggested that 3D differentiation that was preceded by cell aggregation promoted faster maturation, as indicated by earlier spontaneous contraction, reduced proportion of (proliferative) Ki67^+^ CMs and altered gene expression. More recently, Prajapati *et al.* also compared differentiation protocols that differ widely in the use of small molecules and growth factors, proprietary differentiation media, co-culture with End-2 endodermal cells and in 2D vs. 3D culture (Prajapati et al., 2021). Single cell patch-clamp technique showed that hPSC-CMs generated using 3D differentiation had greater maximal upstroke velocity (dV/dtmax) than 2D monolayer cultures, while other electrophysiological parameters including beating rate, action potential amplitude, and maximum diastolic potential were similar. Overall, there is still no clear consensus about the effect of 2D vs. 3D differentiation on maturation.

Compared to the various reports comparing CMs generated using 2D- and 3D-methods, there have been few reports on the characterization of CMs generated using different 2D monolayer protocols. Here we defined the temporal control of the WNT signaling pathway to be a determinant not only of differentiation efficiency, but also of the maturation stage of the resultant cells. Specifically, we demonstrated differential structural, mitochondrial/metabolic and proliferative properties in hPSC-CMs generated using different differentiation schedules. It is well known that cardiac differentiation requires very precise control of the WNT signaling pathway, and that inappropriate activation/repression can reduce differentiation efficiency, as we also showed here with the “2 + 2 + 2” schedule. By contrast, the role of the WNT signaling pathway on cardiac maturation is less clear. Recent studies by Buikema *et al.*, Fan *et al.*, and Quaife-Ryan *et al.* all showed that activation of the WNT signaling pathway can promote proliferation of hPSC-CMs (Fan et al., 2018; Buikema et al., 2020; Quaife-Ryan et al., 2020). Since continued proliferation is often considered as a sign of immaturity, sustained proliferation may indicate the maintenance of hPSC-CMs at an immature state. To test this, Buikema *et al.* evaluated the behavior and transcriptomic profile of hPSC-CMs treated with CHIR-99021, which could activate WNT signaling by inhibiting GSK-3β (Buikema et al., 2020). Treated CMs had less organized sarcomeric structure and reduced expression of genes associated with developmental maturity, thereby showing that the WNT signaling pathway is a negative regulator of cardiac maturation. It is important to note that the above experiments were done on committed CMs, while our study involved the modulation of the WNT signaling pathway during cardiac differentiation. Nonetheless, extrapolating from these results, it is possible that inhibition of WNT signaling pathway at a later stage of differentiation (from D3-5 in the “1 + 2 + 2” schedule, compared to D2-4 of the “2 + 2” schedule) may affect the maturation state of CMs.

Moreover, we performed bioinformatics analysis to show that select cardiac transcription factors and miR-200c may contribute to the mRNA differences observed between our “1 + 2 + 2” and “2 + 2” cultures. Our result demonstrates that the interplay of TFs and miR-200c could play a role in the differential regulation of genes important for differentiation and maturation among our cultures.

In summary, we optimized a cardiac differentiation protocol that is robust, cost-effective, highly efficient, and promotes a more developmentally advanced phenotype. The successful use of hPSC-CMs for disease modeling and cardiotoxicity screening depends on their ability to recapitulate the properties of their adult counterparts. Our simple protocol will facilitate the production of highly pure, mature CMs for research and therapy.

## Data Availability

The original contributions presented in the study are included in the article/Supplementary Material, further inquiries can be directed to the corresponding author.
