# Supplementary material for: Temporal Control of the WNT Signaling Pathway During Cardiac Differentiation Impacts Upon the Maturation State of Human Pluripotent Stem Cell Derived Cardiomyocytes
Source: Front Mol Biosci. 2022 Mar 24;9:714008. doi: 10.3389/fmolb.2022.714008 (PMC8987729; doi:10.3389/fmolb.2022.714008)
Supplement: Supplementary file 1 [file DataSheet2.pdf]

Supplementary Table 1. Primer sequences used for qRT-PCR

Primer	Primer Sequence (5' – 3')	
B2M	CCACTGAAAAAGATGAGTATGCCT	CCAATCCAAATGCGGCATCTTCA
TNNI3	CCTCAAGCAGGTGAAGAAGG	CAGTAGGCAGGAAGGCTCAG
TNNT2	AGCATCTATAACTTGGAGGCAGAG	TGGAGACTTTCTGGTTATCGTTG
TNNT1	CAGCTCCACGAGGACTGAAC	CTCTTCAGCAAGAGTTTGCG
MYH7	GAGGACAAGGTCAACACCCT	CGCACCTTCTTCTCTTGCTC
MYL7	CCGTCTTCCTCACGCTCTT	TGAACTCATCCTTGTTCACCAC
MYL2	ACATCATCACCCACGGAGAAGAGA	ATTGGAACATGGCCTCTGGATGGA
CD36	CCACAGAAGGATACAGGACAAA	CATTTACTATGGCGGCTATCA
COX6A2	GAGTTCCGTCCCTACCAACA	CAGAGGGTTCACGTGGCTAT
COX7B	CACCTTCACGATGTTTCCCT	AGGTGTACGTTTCTGGTGGC
MT-ATP6	CAACAACCGACTAATCACCA	GGGTGGTTGGTGTAAATGAG
KCNJ2	CTTGGGAATTCTGGTTTGCT	TGACTCAGCTGACATCCACAC
HCN1	ACGGTGTTGCTGGTGTCATT	TCAGCAGGCAAATCTCTCCAA
CACNA1C	TTCGTCATCGTCACCTTTCAG	TGTACTGGTGCTGGTTCTTG
ATP2A2	CAATGGCGCTCTCTGTTCTA	ATCCTCAGCAAGGACTGGTT
NR2F2	TCACCCGCCAAACTAAAGGA	CTCTGCACCGCAAAACCATA
KCNA5	GTAACGTCAAGGCCAAGAGC	TCCCATTCCCTACTCCACTG
NPPA	ATGAGCTCCTTCTCCACCAC	TCCAGCAAATTCTTGAAATCC
TBX3	TGAAGACCATGGAGCCCGAA	GAGGAAACATTCGCCTTCCCG
SHOX2	CGACTGACGGAGGGTAGAA	ACACAAGAAACCGAAACGCCT
HCN4	ACCGCTATCAAAGTGGAGGG	GGTCAGGTCCCAGTAAAATCTGA
