# Supplementary material for: Temporal Control of the WNT Signaling Pathway During Cardiac Differentiation Impacts Upon the Maturation State of Human Pluripotent Stem Cell Derived Cardiomyocytes
Source: Front Mol Biosci. 2022 Mar 24;9:714008. doi: 10.3389/fmolb.2022.714008 (PMC8987729; doi:10.3389/fmolb.2022.714008)
Supplement: Supplementary file 2 [file DataSheet1.pdf]

Supplementary Figures

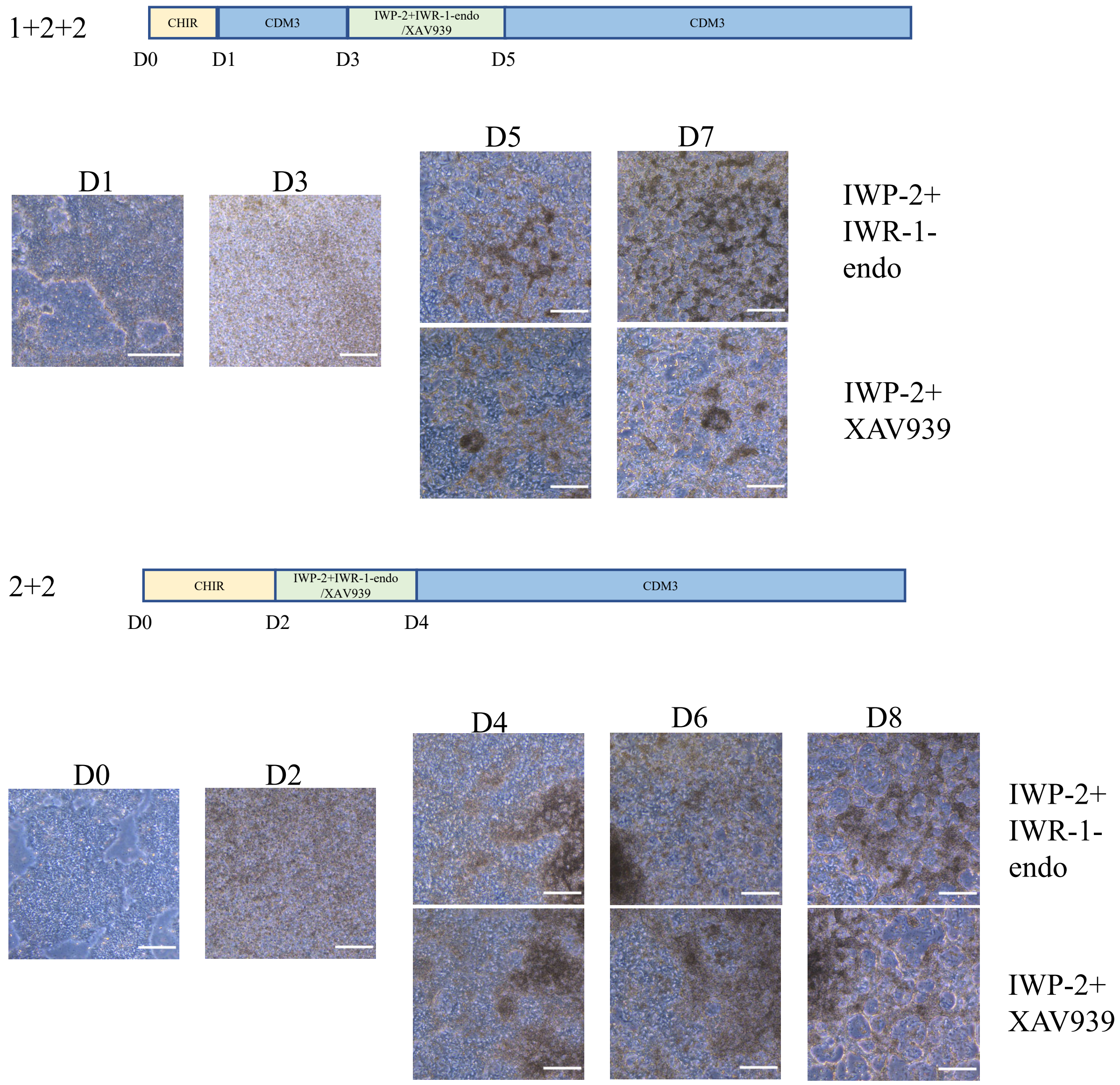

Supplementary Figure 1. Morphological characterization of cardiac differentiation. Representative images of MDI-C16 hPSC, differentiated using the 1+2+2 and 2+2 protocols with two combinations of WNT inhibitors (IWP-2+IWR-1-endo/XAV939). Scale bar, 250 µm.

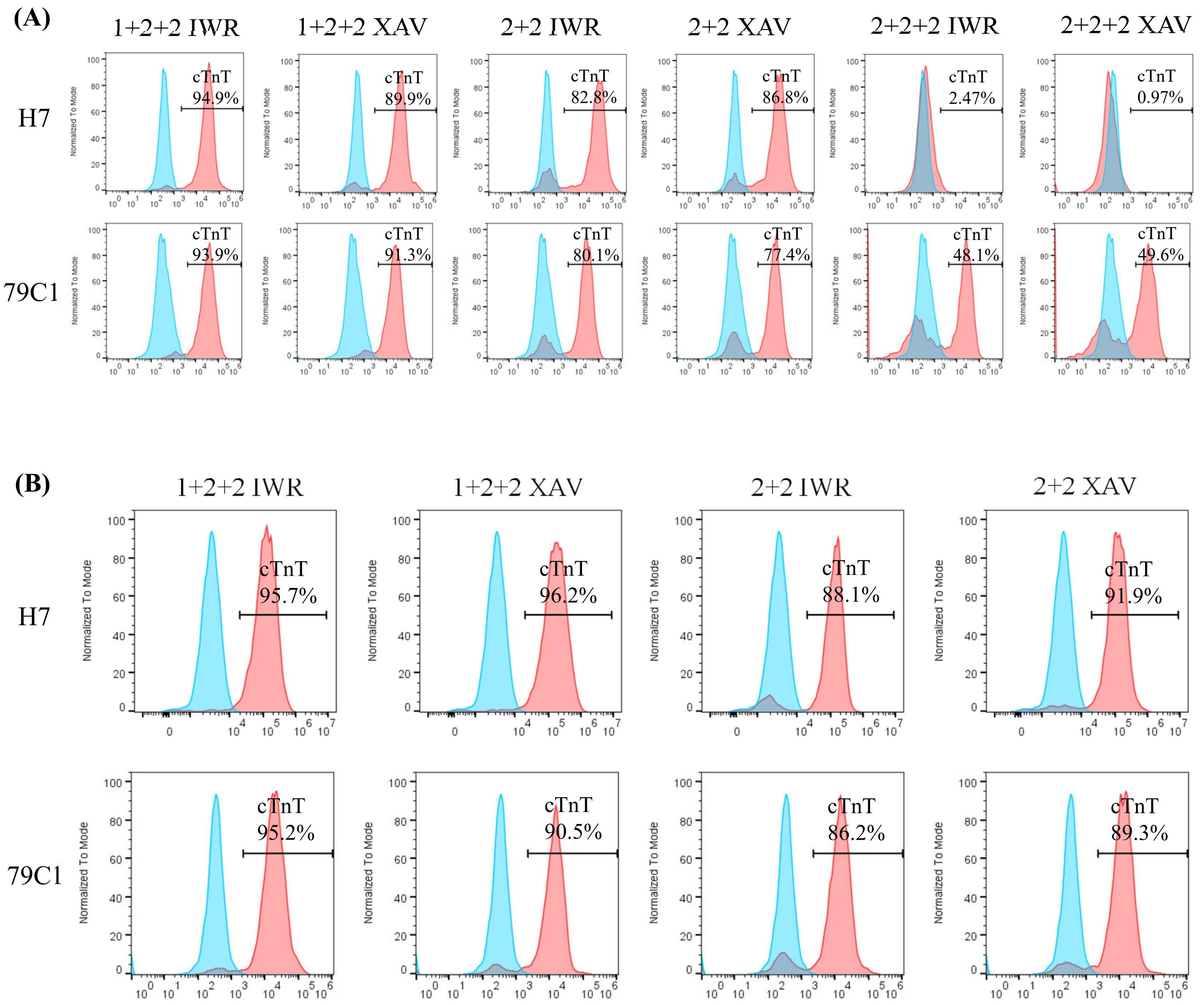

Supplementary Figure 2. Cardiac differentiation efficiency. Representative flow cytometric analysis of TNNT2$^+$ cells in H7 and 79C1 hPSC-CM cultures on **(A)** day 15 and **(B)** day 30 of differentiation. Blue and red histograms represent isotype and TNNT2 staining respectively. (n=5).

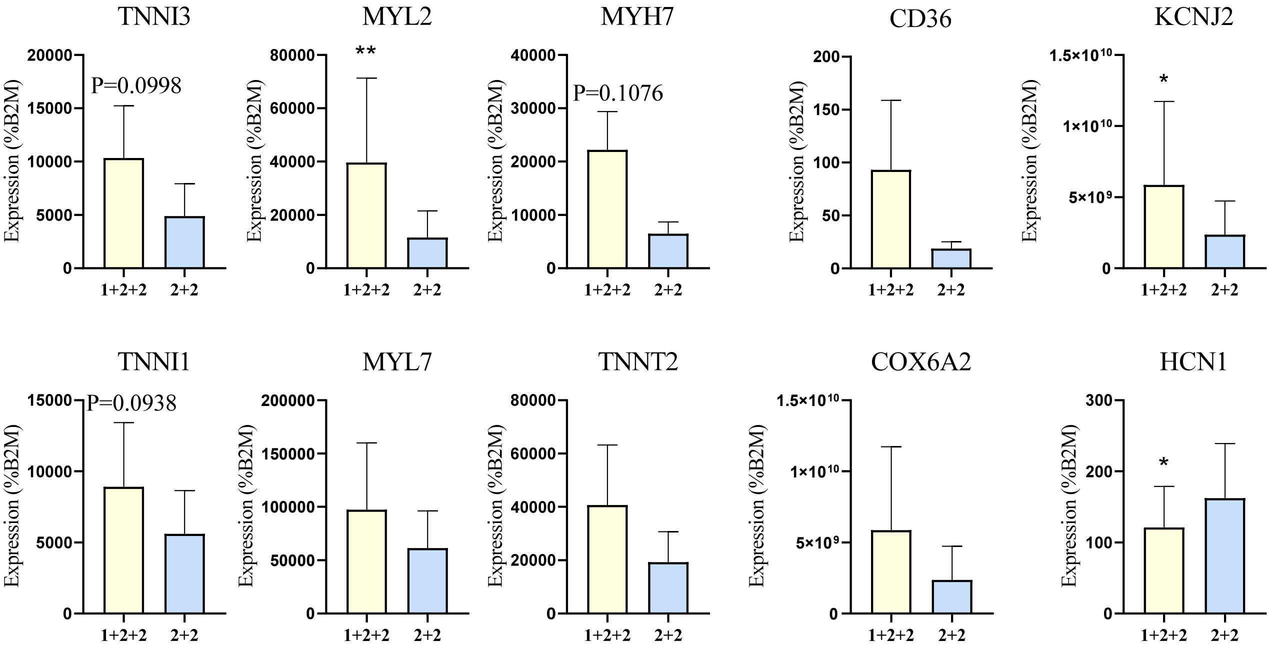

Supplementary Figure 3. Expression of genes important for cardiac maturation. q-PCR analysis of 79C1 hPSC-CMs on day 30 of differentiation. (n=3-4).

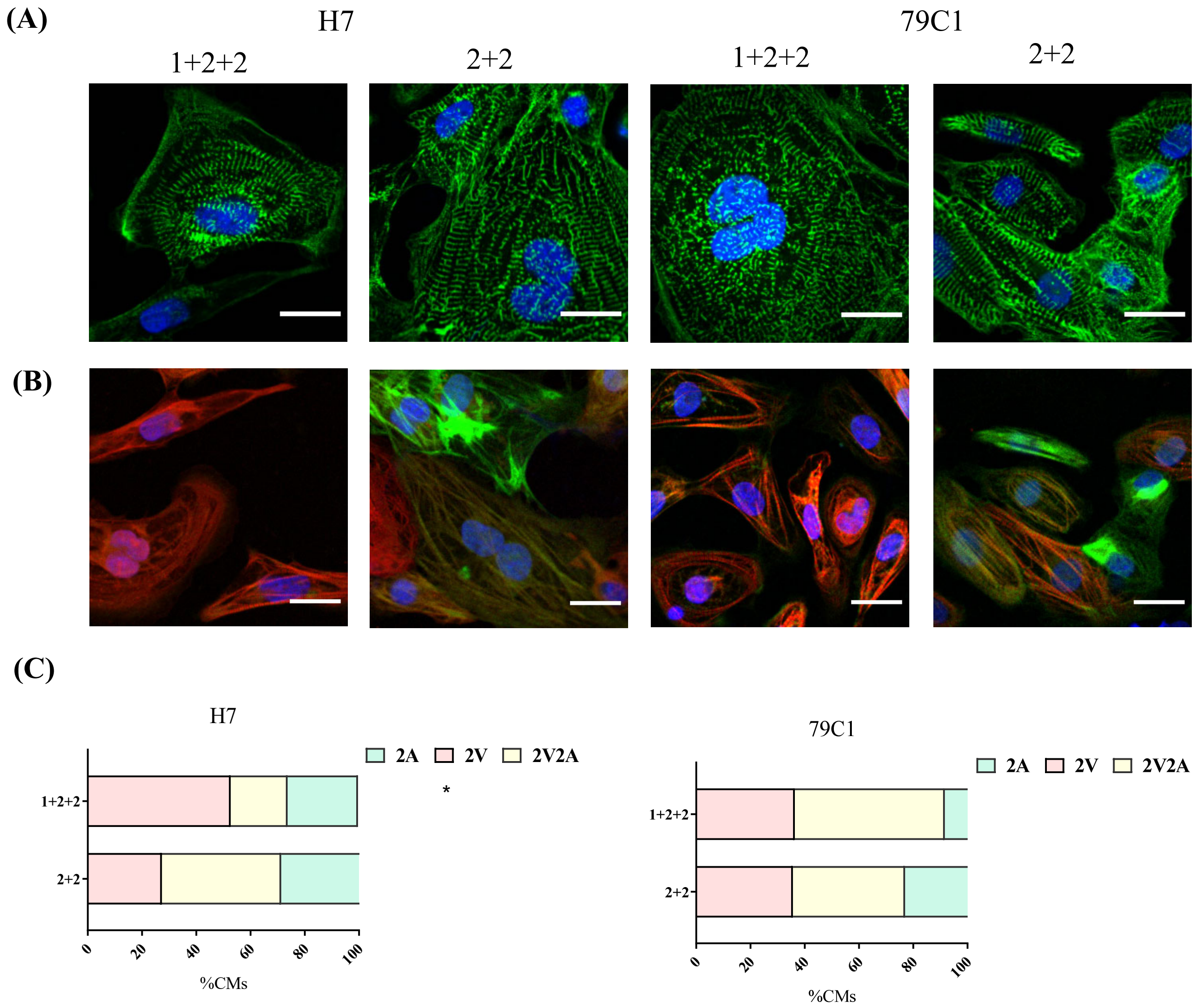

Supplementary Figure 4. Structural characterization. Confocal images of **(A)** α-actinin (green) **(B)** MLC2A (green) and MLC2V (red) in H7 and 79C1 hPSC-CMs generated using selected differentiation protocols, on day 30 of differentiation (n=3). Scale bar, 20 µm. **(C)** The proportion of $MLC2A^{+}$, $MLC2A^{+}$ $MLC2V^{+}$ and $MLC2V^{+}$ cells were quantified (n=3).

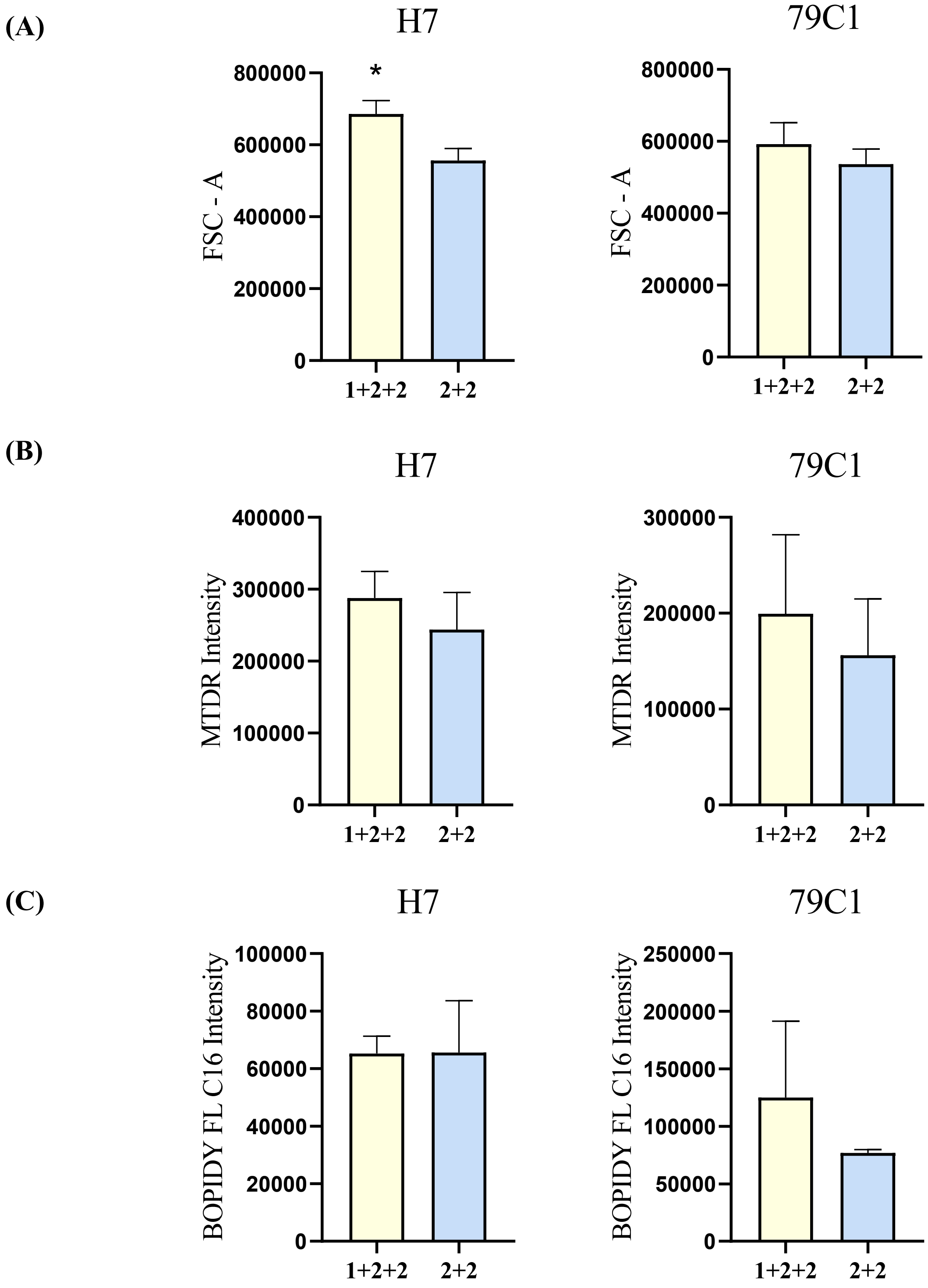

Supplementary Figure 5. Metabolic characterization. **(A)** Forward-scatter (FSC) was measured as a surrogate of cell size in H7 and 79C1 hPSC-CMs derived from selected differentiation protocols on day 30 of differentiation. (n=3). **(B)** Mitochondrial abundance was measured using MitoTracker Deep Red staining. (n=3). **(C)** Fatty acid uptake was monitored using BODIPY FL C16 staining. (n=3).
